# A Sandwich Nanostructure of Gold Nanoparticle Coated Reduced Graphene Oxide for Photoacoustic Imaging-Guided Photothermal Therapy in the Second NIR Window

**DOI:** 10.3389/fbioe.2020.00655

**Published:** 2020-06-30

**Authors:** Zhihua Wang, Xiao Sun, Ting Huang, Jibin Song, Yudong Wang

**Affiliations:** ^1^Department of Gynecology, International Peace Maternity and Child Health Hospital, Shanghai Jiao Tong University School of Medicine, Shanghai, China; ^2^Shanghai Municipal Key Clinical Specialty, Shanghai, China; ^3^Shanghai Key Laboratory of Embryo Original Disease, Shanghai, China; ^4^MOE Key Laboratory for Analytical Science of Food Safety and Biology College of Chemistry, Fuzhou University, Fuzhou, China

**Keywords:** gold nanoparticle, reduced graphene oxide, photoacoustic imaging, the second near-infrared window, photothermal therapy, ovarian cancer

## Abstract

We explore a sandwich-type gold nanoparticle coated reduced graphene oxide (rGO-AuNP) as an effective nanotheranostic platform for the second near-infrared (NIR-II) window photoacoustic (PA) imaging-guided photothermal therapy (PTT) in ovarian cancer. The PEG was loaded onto the AuNPs surface to increase the stability of nanostructure. The forming rGO-AuNPs- PEG revealed very strong SERS signal, NIR-II PA signal and high photothermal efficiency against tumor upon 1,061 nm laser irradiation. The prominent performance was attributed to the plasmonic coupling of AuNPs, and the enhanced response of rGO and the plasmonic AuNP. Thus, our study demonstrates that the rGO-AuNP nanocomposite could promise to be a potential photothermal agent and pave the way for the diagnosis and therapy of ovarian cancer in the future.

## Introduction

Ovarian cancer has the highest mortality among all gynecological cancers (Kossaï et al., 2018; Gao et al., 2019). Despite the remarkable progresses in ovarian cancer therapeutics including conventional surgical resection, radiotherapy and chemotherapy and etc., prognosis remains very poor due to severe adverse reactions and unsatisfactory treatment outcomes (Reimer et al., 1977; Narod, 2016; De Felice et al., 2017; Donnez and Dolmans, 2017; Trimbos, 2017). Therefore, tremendous efforts in biomedical research have been devoted to developing more accurate and effective strategies for diagnosis and therapies of ovarian cancer (Nukolova et al., 2011; Romero and Bast, 2012; Liu and Matulonis, 2014; Nick et al., 2015; Grunewald and Ledermann, 2017; Schwartz et al., 2018; Wang et al., 2018).

Recently, the photoacoustic (PA) imaging-mediated photothermal therapy (PTT) is an emerging treatment, which can potentially improve therapeutic efficacy against cancer (Huang et al., 2014; Chen et al., 2019; Jin et al., 2019; Xu et al., 2019). PTT utilizes the photothermal effect of photothermal conversion agents that can convert light energy into heat by locally activated upon skin-penetrating NIR radiation (Liu et al., 2019), which raise the temperature of surrounding tissue and trigger the death of cancer cells. More notably, PTT is a highly efficient and non-invasive and harmless therapeutic technique (Kim et al., 2009; Zhang et al., 2019). PA imaging is a novel biomedical imaging modality, which integrates optical illumination and ultrasound (US) detection (Ntziachristos and Razansky, 2010). In principle, upon pulsed laser irradiation, the molecules absorb light and converted to heat, then generating an acoustic wave because of thermoelastic expansion (Wang, 2009; Wilson et al., 2013; Deán-Ben et al., 2017; Attia et al., 2019). PA imaging enables multiscale imaging of biological structures with high resolution and deep tissue penetration (Mallidi et al., 2009; Wang and Hu, 2012; Zou et al., 2017) and real-time guides the operation of surgery by providing instant diagnostic functions (Liu et al., 2015; Song et al., 2017). Therefore, the theranostic platforms for simultaneous of PA imaging and PTT have been prepared (Guo et al., 2017; Dong et al., 2018; Gong et al., 2018; Tsai et al., 2018; Yang et al., 2018; Wang et al., 2019). In the past decades, significant advances in NIR light-mediated nanoplatforms ranging from inorganic materials to organic materials have rapidly promoted the developments of phototheranostics for biomedical applications (Ng and Zheng, 2015; Weber et al., 2016; Du et al., 2017; Cai et al., 2018; Yang and Chen, 2019; Yin et al., 2019; Zhu et al., 2019). Among them, reduced graphene oxide (rGO) with a large surface area has been widely explored as nanocarriers of drug and gene delivery (Kim and Kim, 2014; Chen et al., 2016; Nejabat et al., 2017). Besides, the intrinsic NIR absorption allows rGO to be used as PA and PTT contrast agents for theranostic applications (Sheng et al., 2013; Moon et al., 2015; Orecchioni et al., 2015; Song et al., 2015; Hu et al., 2016). However, the rGO has a broad absorption spectrum from the UV to NIR region and low quantum efficiency, which results in relatively low photothermal conversion efficiency (Zhu et al., 2010). It was reported that the photothermal performance of rGO can be improved by conjugation with plasmonic nanoparticles (Moon et al., 2015; Song et al., 2015; Lin et al., 2016).

**Scheme 1:**
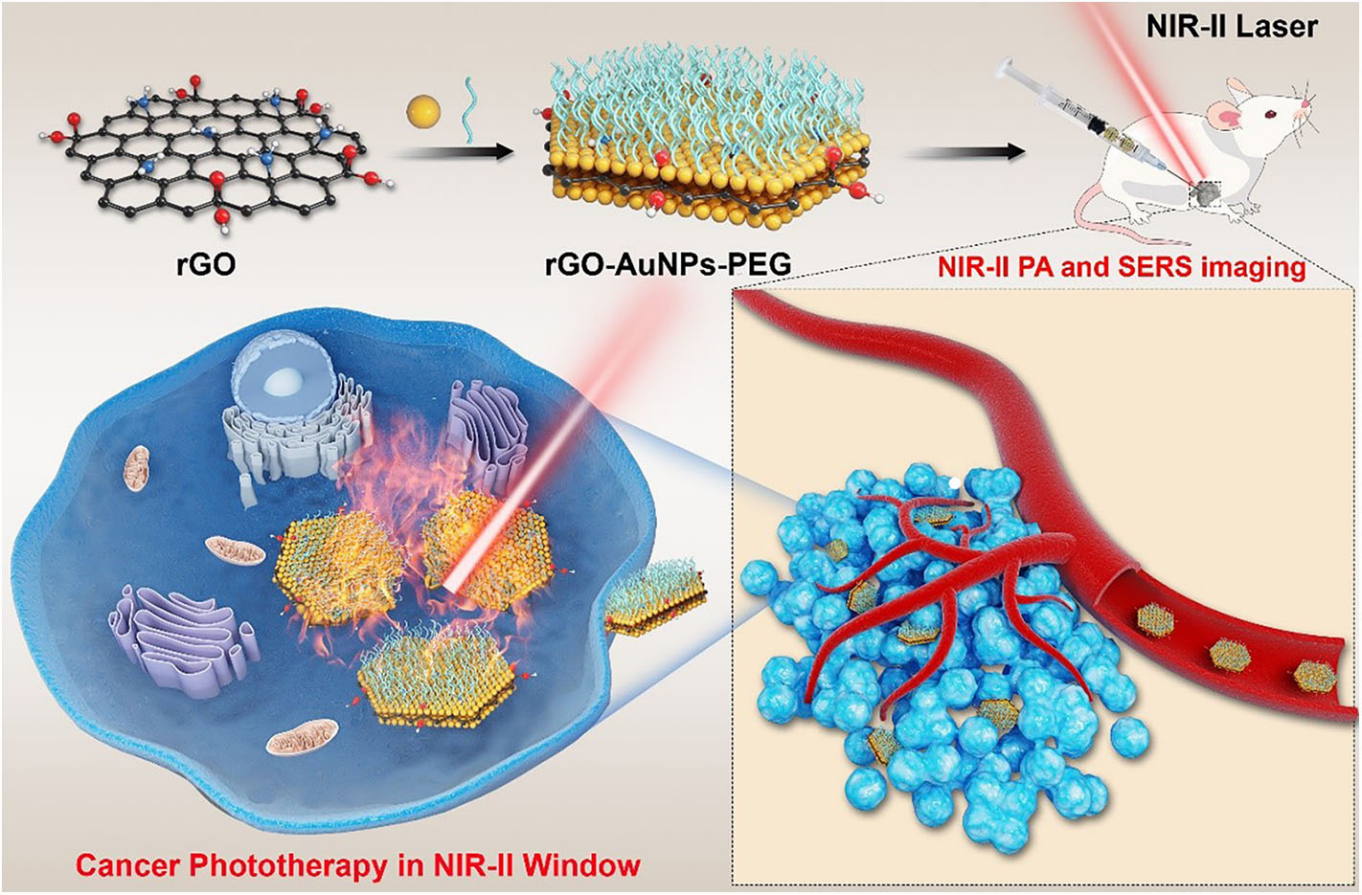
Schematic illustration of the rGO-AuNP with high SERS signal, NIR-II photoacoustic and photothermal properties used for cancer therapy in the second window.

Herein, we have developed the sandwich-type rGO-AuNP nanocomposite as an enhanced theranostic nanoplatform for NIR-II photoacoustic imaging and PTT in diagnosis and therapies of ovarian cancer, as shown in Scheme 1. The photothermal effect of the rGO-AuNP nanoform in the NIR-II window was greatly enhanced compared with AuNPs or rGO, due to the strong plasmonic coupling between the AuNPs and the generated electromagnetic filed increased the light absorption efficiency of the rGO. Moreover, the surface enhanced Raman scattering (SERS) signal of the rGO was also greatly enhanced due to the plasmonic effect between two layers of the AuNPs. High resolution NIR-II PA imaging of the tumor was obtained because the high accumulation efficiency of the rGO-AuNP in the tumor region, providing rich information of the tumor, such as tumor size, localization, morphology NIR-II PA imaging was further used to guide cancer PTT in the NIR region, showing high therapeutic effect. Because the rGO-AuNP was dissociated into single AuNPs and rGO after laser irradiation, it was quickly removed from the body after cancer imaging and therapy, thus greatly reducing the side effect to the body. Overall, the rGO-AuNP nanocomposite was an excellent NIR-II phototheranostic nanoplatform.

## Materials and Methods

### Materials

Silver hexafluorophosphate (98%), potassium hexafluorophosphate (99.5%), N, N, N′, 2-hydroxyethyl disulfide, α-bromoisobutyryl bromide (98%), anisole, tetrafluoroboranuide (IR 1061, 80%), hydrogen tetrachloroaurate (III) trihydrate (HAuCl_4_·3H_2_O), and sodium borohydride (96%) silver nitrate (99%) were purchased from Sigma Aldrich. Poly (ethylene glycol) methyl ether thiol (SH-PEG, average Mw 5000) was purchased from Ruixi Biological Technology (Xi'an, China). Ultrapure water (18.25 MΩ resistivity, 25°C) was used in all experiments. Propidium iodide (PI) dye and Annexin V-FITC apoptosis detection kit were purchased from Beyotime Biotechnology (Shanghai, China). Cell Counting Kit-8 (CCK-8) was purchased from MedChemExpress (Monmouth Junction, NJ, USA).

### Preparation of the rGO-AuNP

Au NPs were firstly synthesized through the citrate reduction method (Turkevich et al., 1951; Frens, 1973). Graphene oxide (GO) wasproduced from graphite (1 g) by a modified Hummers method using NaNO_3_, KMnO_4_, and H_2_SO_4_. After purification for three times, 10 mg of the as-prepared GO was dispersed in 100 mL of DI water and sonicated for 20 min, and then 100 mg L-cysteine was injected into the GO solution. The pH value of the solution was tuned to 10.0 using NaOH (1M) solution. The mixed solution was then heated to 75°C for 2 h. After purification by centrifugation, the as-prepared L-cysteine conjugated GO (40 mg) was reduced by the hydrazine solution (0.3 mL, 65 wt% in water) at 90°C for 1 h. To further immobilize AuNPs on L-cysteine conjugated GO surface, 10 mL of the L-cysteine conjugated GO solution and AuNPs solution (50 mM, 10 mL) were mixed together and sonicated for another 1 h. After adding PEG-SH, the solution was further sonicated for 0.5 h to form rGO-AuNP.

### Photothermal Effect of the rGO-AuNP

Four hundred μL of rGO-AuNP nanocomposites in 1 mL eppendorf vials were irradiated with a 1,061 nm diode laser (spot size: 1 cm) with different power densities for 5 min, respectively. Real-time temperature elevation and thermographic images of the samples in the aqueous solution were recorded by an infrared thermographic camera as a function of laser irradiation time. PBS was employed as a negative control sample.

### *In vivo* NIR-II Photoacoustic Imaging of rGO-AuNP Irradiated With NIR-II Laser

For *in vivo* NIR-II photoacoustic imaging, SKOV-3 tumor bearing mice were used for imaging experiments. The experiments involving animals were approved by the Ethics Committee of International Peace Maternity and Child Health Hospital of China. The SKOV-3 tumor was induced by inoculating the SKOV-3 cancer cells (1 × 10^6^ cells in 100 μL PBS) into the right shoulder of nude mice (6 week-old females) under anesthesia. After 14 days, the samples in the PBS solution (200 μL, 1 mg/mL) were then intravenously injected into the SKOV-3 tumor-bearing nude mice. Meanwhile, the entire tumor region of these mice was scanned using a VisualSonic Vevo 2100 LAZR system equipped with a 40 MHz, 256-element linear array transducer as a function of time. The NIR-II PA imaging was acquired by using 1,061 nm laser and the image was constructed by using VisualSonic Vevo software. During the test, the mice was anesthetized by using Isoflurane along with oxygen using an anesthesia system.

### *In vivo* SERS Imaging Test

SERS detection and imaging of the tumor was obtained after intravenous injection of the rGO-AuNP samples. The line-shaped Raman laser spot was irradiated on the tumor via a combination line scanning optics and cylindrical lens. Raman signal was then tested by using a 10 × objective (NA = 0.3). The line-shaped Raman laser was scanned by using galvanometer mirror at the tumor plane. The used laser power is 0.2 mW μm^−2^ and the irradiation time for each line is 40 s.

### *In vivo* Photothermal Cancer Therapy in the NIR-II Region

For tumor NIR-II PTT therapy, the samples in PBS (200 μL) were IV injected into the SKOV-3 tumor-bearing mice under anesthesia. The 1,061 nm laser irradiation of the tumor region was processed at 24 post-injection due to the high accumulation efficiency at the time point. Moreover, thermal images of the whole tumor region were recorded during the irradiation using a SC300 infrared camera (FLIR). Meanwhile, the average temperature variation of the whole tumor region was calculated using the FLIR analyzer software. After the tumor was treated with different samples and laser irradiation, the dimension of the tumor was measured using a caliper at different time points. The tumor volume V (mm^3^) was then analyzed using the formula: V = LW^2^/2, where W and L are the width and length of the tumor.

## Results and Discussion

### Preparation and Characterization of the rGO-AuNP

**Figure 1:**
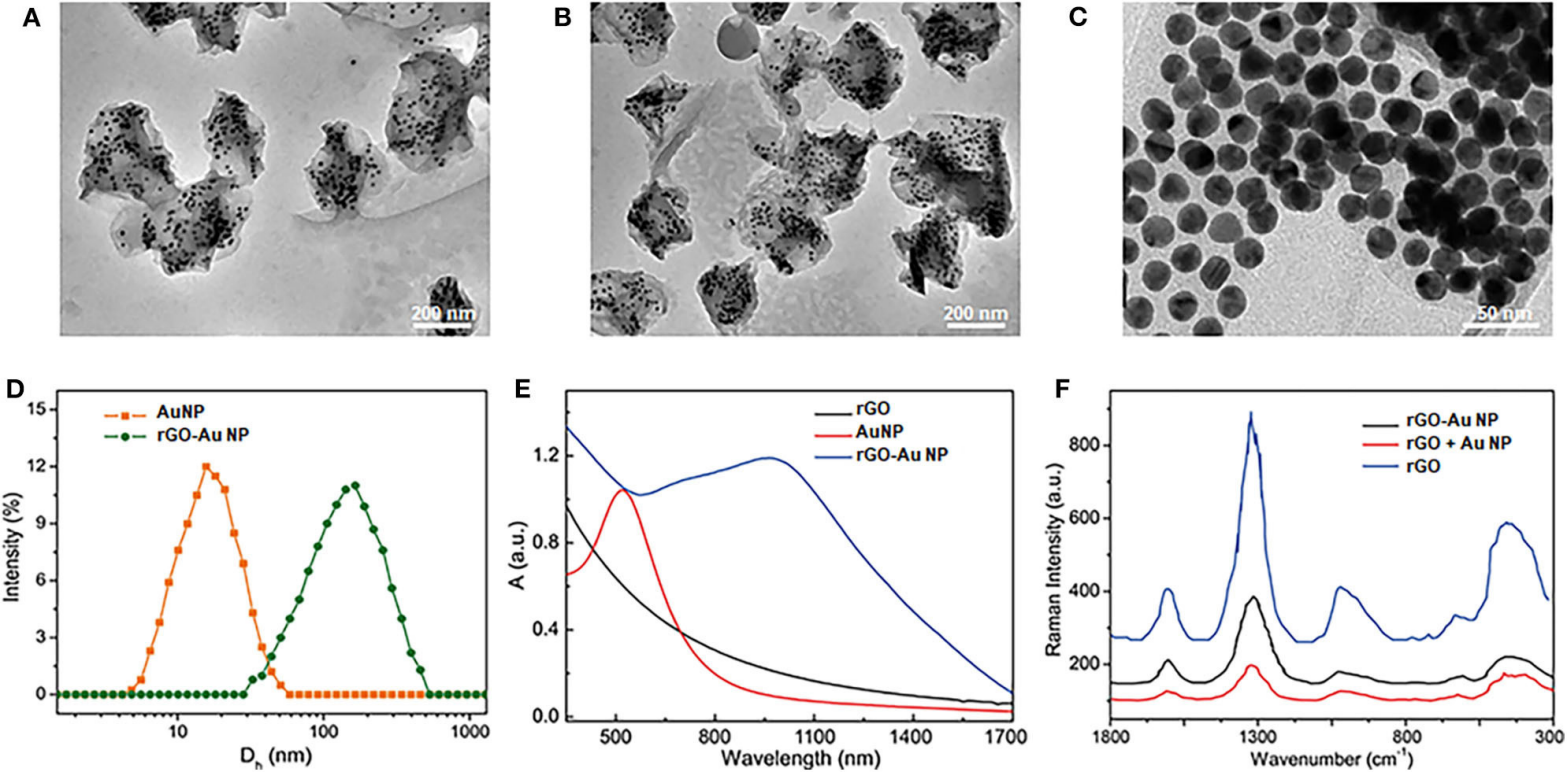
Characterization of the theranostic rGO-AuNP nanocomposite. **(A–C)** TEM images of rGO-AuNP at different densities and magnifications. **(D)** Hydro dynamic distribution of the AuNPs and rGO-AuNP. **(E)** UV-vis spectra of the rGO-AuNP. **(F)** SERS spectra of the rGO, the mixture of AuNPs and rGO, and rGO-AuNP complex nanosheet.

Graphite oxide (GO) was first prepared from graphite by a modified Hummers method through employing NaNO_3_, H_2_SO_4_, and KMnO_4_. To modify AuNPs on the GO surface, L-cysteine was then modified on the surface of GO in aqueous surface. After purification of the samples, AuNPs with the size of 14 nm was conjugated on the GO surface through covalent Au-S bond (Figure S1). Benefit from the high density of thio groups, AuNPs were closely arranged on the GO surface. The GO was further reduced to rGO by hydrazine to increase its light absorption efficiency. To increase the stability, poly(ethylene glycol) (PEG) with MW = 5,000 was modified on the AuNPs surface, forming theranostic rGO-AuNPs, as shown in the transmission electron microscopy (TEM) images at different densities and magnifications (Figures 1A–C). The average size of the rGO-AuNP was 160 nm, showing obvious size increase compared with AuNPs, as displayed in Figure 1D. We observed a lot of gold nanoparticles were coated onto double sides of the reduced graphene oxide. Moreover, the rGO-AuNP presented a sandwich nanostructure, which will be in favor of enhancing the subsequent photothermal and photoacoustic effects. In comparison with the AuNPs, the absorption peak of rGO-AuNP showed great red-shift and had strong light absorption in the NIR-II region (Figure 1E). It was because the strong plasmonic coupling between AuNPs on the rGO surface. Moreover, the electromagentic field generated by the AuNPs further enhanced the light absorption efficiency of the rGO.

Moreover, because strong plasmonic effect between the two layers of the AuNPs, greatly enhanced electromagnetic filed was distributed in the embedded rGO layer, which great enhanced the surface enhanced Raman signal (SERS) of rGO, as shown in Figure 1F. The Raman enhanced factor of the rGO-AuNP was calculated as 5 × 10^7^, which could be served as an SERS probe for the cancer cell detection of *in vivo* Raman imaging of the tumor. The enhanced electromagnetic field of the rGO-AuNP is also very benefit for its physical-optical properties, such as increasing the photo conversion efficiency under laser irradiation.

### Enhanced Photothermal and Photoacoustic Properties of the rGO-AuNP

**Figure 2:**
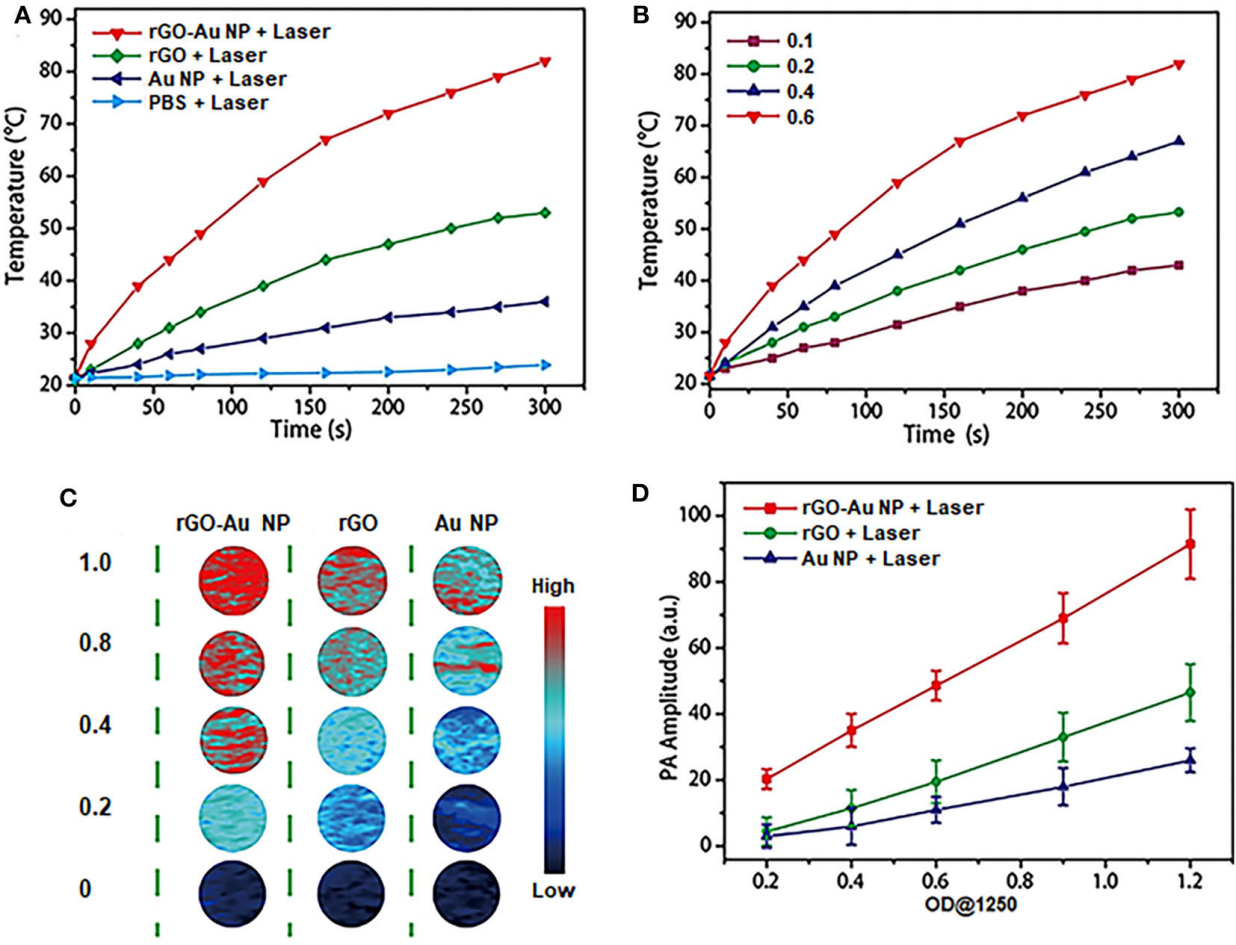
NIR-II photothermal and photoacoustic properties of rGO-AuNP nanocomposite. **(A)** Temperature variations of PBS, rGO, the mixture of rGO and AuNP and rGO-AuNP irradiated with a 1,061 nm laser at a power density of 0.5 W/cm^2^ as a function of PEG irradiated with a 1,061 nm laser at a power density of 0.5 W/cm^2^ as a function of time. **(B)** Temperature increase curves of the rGO-AuNP at different concentrations irradiated with laser. **(C)** Photoacoustic images of rGO, AuNPs, and rGO-AuNP aqueous solutions at different concentrations. **(D)** PA intensities of rGO, AuNPs, and rGO-AuNP aqueous solutions illuminated with 1,250 nm laser as a function of OD_1250_ values.

To demonstrate the performance of rGO-AuNP to be used as PTT therapy agents and photoacoustic imaging contrast agents. Firstly, we investigated the enhanced photothermal properties of the rGO-AuNP nanocomposite. Figure 2A described the temperature variation curves of the rGO-AuNP and control samples in aqueous solution upon laser irradiation of a 1,061 nm wavelength as a function of irradiation time. No obvious temperature change was observed for PBS solution after laser irradiation for 5 min. The temperature increment of the rGO-AuNP is around 61°C, which is higher than those of the AuNPs (15°C) and rGO (33°C). The result is attributed to the electronic interaction among AuNPs, and between the AuNPs and rGO under laser illumination. As shown in Figure 2B, the temperature of rGO-AuNP solution increased with increasing the concentration, exerting a well-defined concentration-dependent photothermal heating effect.

Moreover, we further studied the PA properties and feasibility by utilizing rGO-AuNP as a PA imaging contrast agent. PA images of the rGO-AuNP and control samples in aqueous solution upon PA laser irradiation of a 1,250 nm wavelength. Figure 2C indicated that PA signals of the rGO-AuNP was stronger than those of the AuNPs and rGO. Quantitative analysis confirmed that the PA intensity of the rGO-AuNP is a much higher than those of the AuNPs and rGO, while the PA intensities of all the samples in aqueous solution increased linearly with increasing OD_1250_ values (Figure 2D). Cumulatively, the results revealed that rGO-AuNP can be a promising NIR-II PA imaging contrast agent.

### *In vitro* Photothermal Therapy by the rGO-AuNP in the NIR-II Region

**Figure 3:**
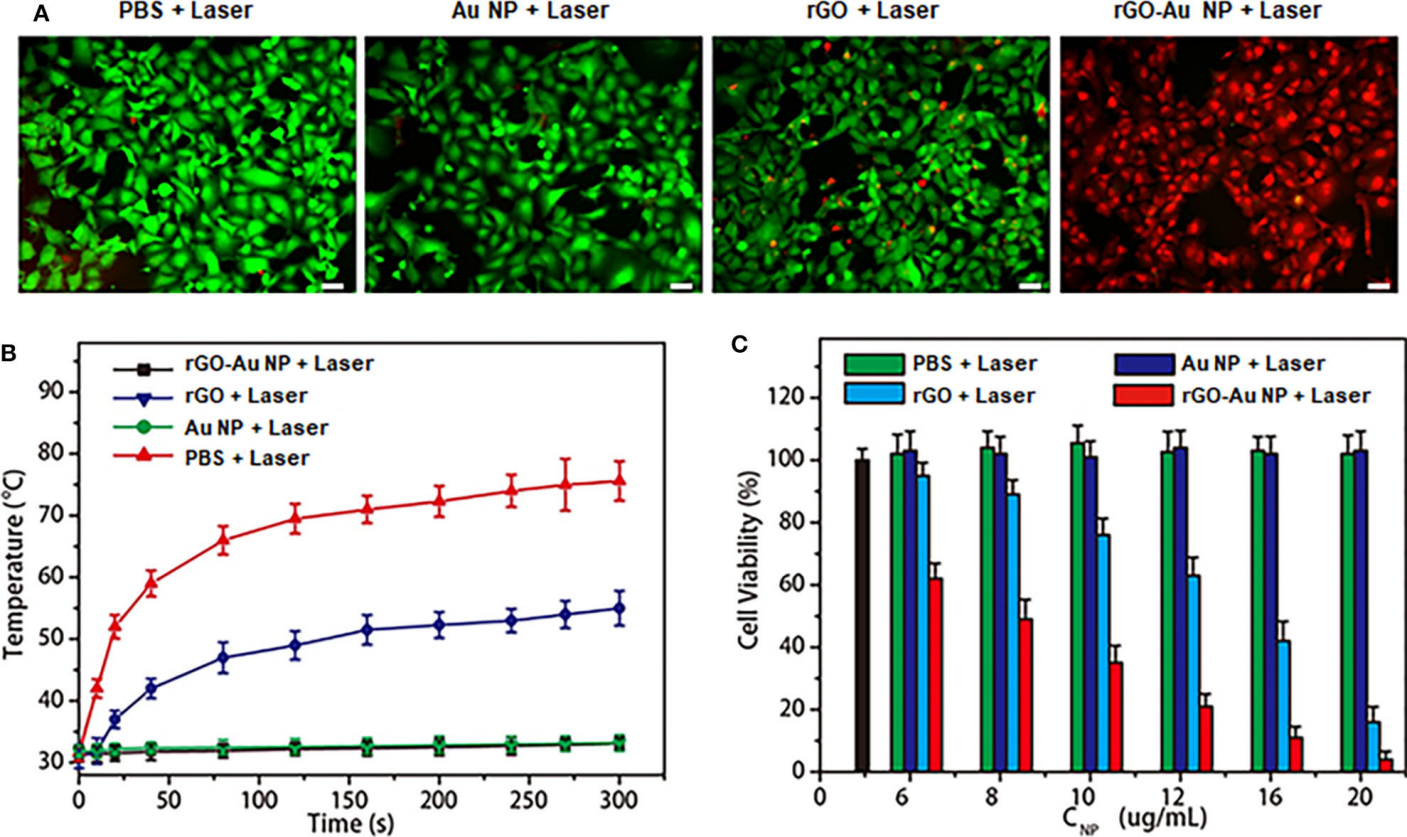
*In vitro* photothermal therapy with tumor cells. **(A)** The fluorescence images of SKOV-3 cells treated with rGO and AuNPs and rGO-AuNP for 24 h before a 1,061 nm laser irradiation applied (0.25 W/cm^2^, 300 s). PBS only as a blank control. (Green fluorescence: Calcein AM, red fluorescence: PI. Scale bar: 20 μm). **(B)** Temperature curves of SKOV-3 cancer cells treated with PBS, rGO, AuNPs, and rGO-AuNP in aqueous solutions irradiated with a 1,061 nm laser as a function of time. **(C)** Cell viability of SKOV-3 cancer cells treated by different nanomaterials at different concentrations and PBS with and without 1,061 nm laser irradiation (Blank bar: control cells).

We next investigated the *in vitro* PTT efficacy and cytotoxicity of the rGO-AuNP. Calcein AM (green) and propidium iodide (PI, red) costaining was used to differentiate the live and dead cells after PTT (Figure 3A). The rGO-AuNP showed high compatibility and safety due to the cell was still very health after incubated with the samples for 48 h at high concentration (Figure S2) for cells. A violent red fluorescence region was observed in the rGO-AuNP group after laser irradiation (1,061 nm, 0.25 W/cm^2^, 300 s), showing that the live cells were completely destroyed. In contrast, control groups of cells treated with rGO and AuNPs exposed to the same laser irradiation, or PBS only displayed widespread green fluorescence but negligible red fluorescence signals, which contributed to minimal cell damage. The temperature curves of SKOV-3 cancer cells treated with PBS, rGO, AuNPs and rGO-AuNP in aqueous solutions upon an 1,061 nm laser irradiation of as a function of irradiation time in Figure 3B. No obvious temperature change was observed for PBS and AuNPs aqueous solutions after laser irradiation for 5 min. The temperature of the rGO-AuNP is much higher than rGO group. An MTT assay was measured to further verify PTT efficacy and cytotoxicity of the rGO-AuNP (Figure 3C). With the concentration increasing of the rGO-AuNP, cell viability of SKOV-3 cells gradually decreased. Meanwhile, the cell viability with the rGO-AuNP is significantly lower than other control groups at the same condition, in accordance with the phenomenon from live/dead assay. Taken together, the results indicated that the rGO-AuNP nanocomposite possesses higher PTT efficacy and more phototoxicity.

### *In vivo* Photoacoustic Imaging in the NIR-II Region

**Figure 4:**
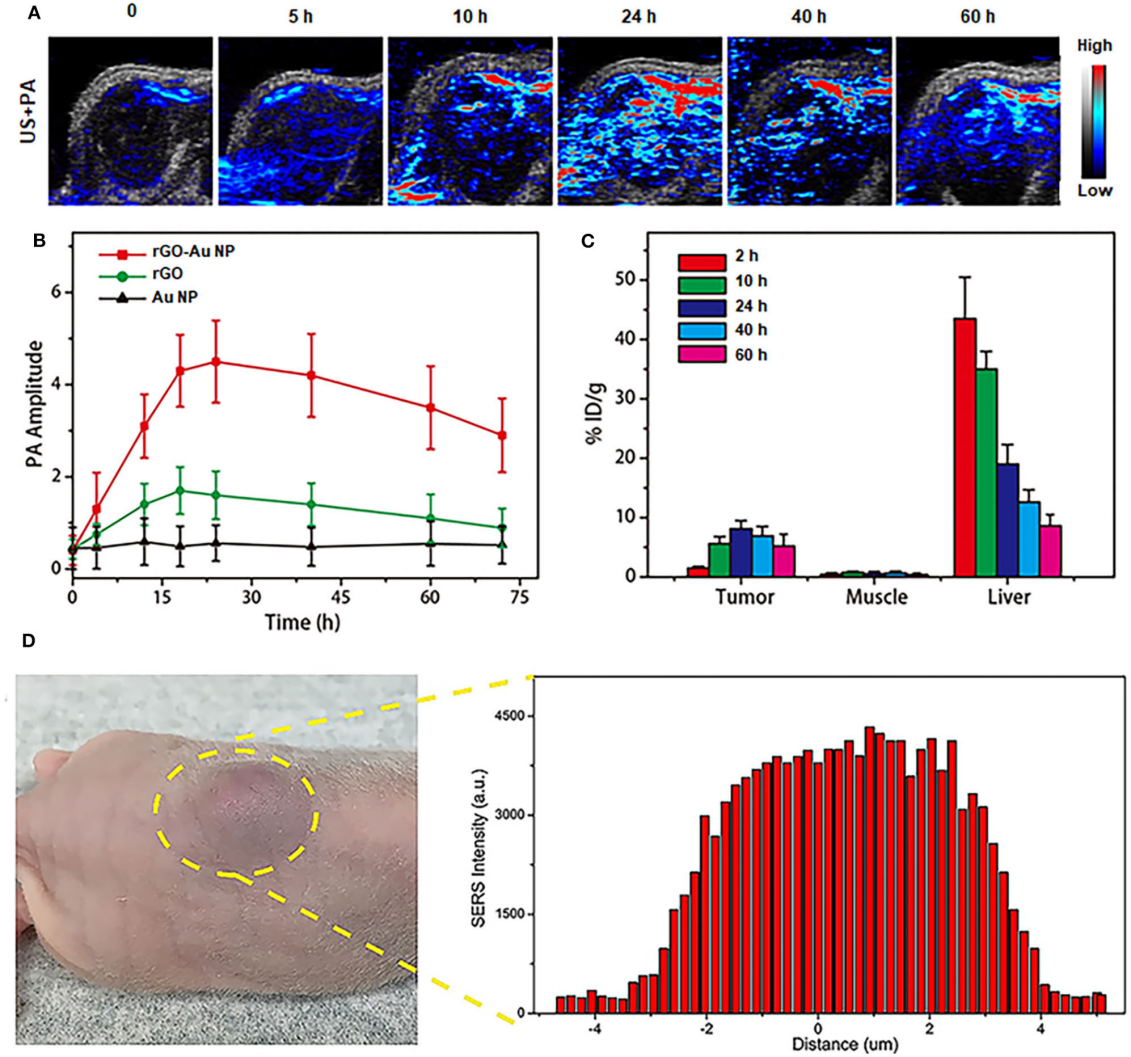
*In vivo* NIR-II PA imaging. **(A)** 2D ultrasonic (US) and PA images of tumor tissues in SKOV-3 tumor-bearing mice treated with rGO-AuNP at different time points post-injection. **(B)** Variations of the PA signal amplitude in the tumor region treated with PBS, rGO, and rGO-AuNP at different time points post-injection. **(C)** Biodistribution of the rGO-AuNP in tumor, muscle and liver at different time points post-injection. **(D)** *In vivo* SERS signal of the tumor and its surrounding tissue to determine the boundary between tumor and normal tissue.

NIR-II PA imaging was employed using rGO-AuNP as a contrast agent to visualize the tumor microstructure and material accumulation in tumor tissues. Thus, the rGO-AuNP was intravenously injected into SKOV-3 tumor-bearing mice, which were then subjected to PA imaging at different time points post-injection (Figure 4A). The PA images of the tumors show that the rGO-AuNP rapidly amassed in the tumor region and obtained the strongest PA signal at 24 h post-injection. More importantly, the PA signal was only observed in the tumor tissue, and no background signal presented in the skin, indicating the higher contrast and resolution of the NIR-II PA imaging of the rGO-AuNP compared with the conventional NIR-I PA imaging. As shown in Figure 4B, we studied the changes of PA signal amplitude in the tumor region treated with PBS, rGO and rGO-AuNP at different time points post-injection. The average tumor PA intensity of rGO-AuNP was higher than control samples, and the intensity derive the maximum value at 24 h corresponding to the phenomenon of the above PA images. *In vivo* biodistribution results confirmed that a large amount of rGO-AuNP accumulated in liver (Figure 4C), while the concentration of rGO-AuNP in the liver gradually reduced over time suggesting a slow clearance rate.

As shown in Figure 4D, SERS signals of the rGO-AuNP at 1,225 cm^−1^ from the SKOV-3 tumor center to its surrounding normal tissue were tested to accurately define the boundaries between them. The disappearance and variation and of these SERS signals was successfully employed to precisely distinguish the SKOV-3 tumor region from normal tissue. Based on the SERS mapping results, the SKOV-3 tumor boundary was defined between defined between 2.9 and 3.8 mm. For the subsequent therapy experiments, especially with the application of PTT *in vivo*, we can control the NIR-II laser spot size to whole cover the tumor region without irradiating the surrounding normal tissue based on the combined NIR-II PA and SERS imaging results.

### *In vivo* Photothermal Therapy Guided by NIR-II PA and SERS Imaging

**Figure 5:**
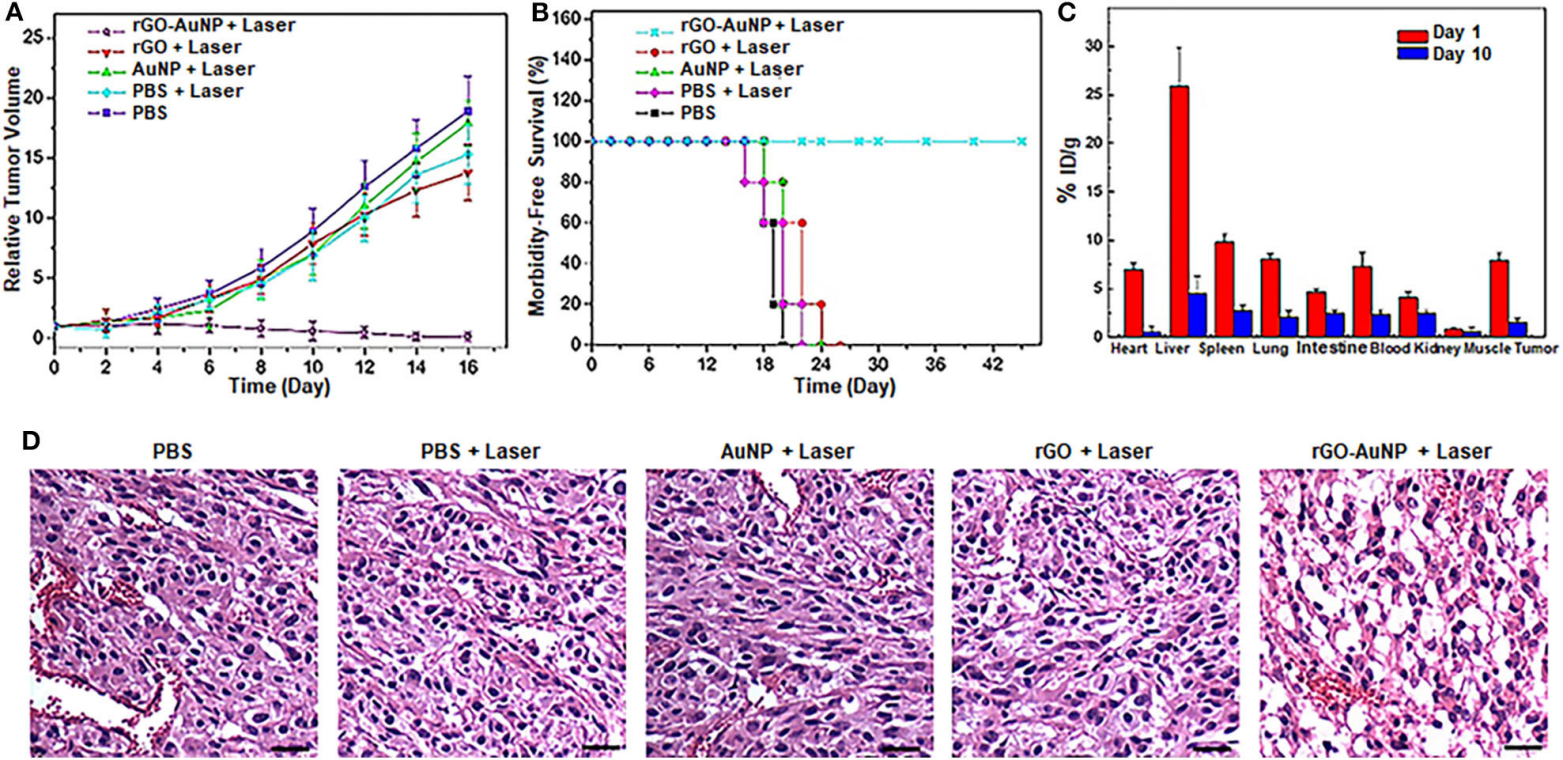
*In vivo* photothermal therapy. **(A)** Relative SKOV-3 tumor volume after various treatments. Tumor volumes were normalized to their initial sizes. **(B)** Survival curves of the SKOV-3-tumor bearing mice after various treatments. **(C)** Biodistribution of the rGO-AuNP in tumor and different organs at day 1 and day 10 post-injection. **(D)** Hematoxylin and eosin (H&E) staining images of the tumor sections treated with different samples. The scale bar is 50 μm.

The *in vivo* photothermal therapeutic effect of the rGO-AuNP nanocomposite was further investigated using NIR-II 1,061 laser. The SKOV-3 tumor bearing mice were randomly divided into five groups with *n* = 5 for each group: (1) control group without any treatment; (2) intravenous injection with PBS and laser irradiation; (3) intravenous injection with rGO and laser irradiation; (4) intravenous injection with AuNPs and with laser irradiation; and (5) intravenous injection with rGO-AuNP and laser irradiation. Tumor volumes and body weights of the mice were continuously monitored for over 2 weeks. As shown in Figure 5A, the tumors in mice treated with group 6 were effectively eliminated without recurrence compared to the continued tumor growth in other control groups. These results indicated that rGO-AuNP presented an excellent photothermal therapeutic effect against tumors. In addition, body weights of the mice had not significantly changes after different treatments in either group (Figure S3). More importantly, mice in the rGO-AuNP survived over 40 days, while mice in other groups showed average life spans of <30 days due to the tumor burden (Figure 5B). It is worth notably is that whether the rGO-AuNP was accumulated in tissues/organs or cleared out of the body after PTT, we collected various tissues/organs containing heart, liver, spleen, lung, kidney, muscle and tumor from the sacrificing mice at 1 day and 10 days post-treatment with rGO-AuNP and NIR irradiation. The relative contents of rGO-AuNP in the various organs and tissues are represented in Figure 5C. After 1 day of the treatments, the rGO-AuNP were mainly accumulated in liver, tumor and spleen. However, after 10 days of the treatments, the levels of rGO-AuNP were obviously reduced or cleared out from all of the organs. As shown in Figure 5D, hematoxylin and eosin (H&E) staining of the tumor tissue after treated with the rGO-AuNP mostly exhibited necrosis areas stained by eosin, which clearly dominated the SKOV-3 tumor tissue. As a whole, the rGO-AuNP as an eminent photothermal probe can be guided photothermal therapy *in vivo* by optical imaging.

## Conclusion

In summary, we have developed an enhanced theranostic platform based on the rGO-loaded plasmonic AuNPs, rGO-AuNP, which presented sandwich nanostructure with excellent photothermal and photoacoustic properties for effective PA imaging and PTT. It was contributed to the response of rGO and the plasmonic gold nanoparticle when irradiated with an NIR laser. The rGO-AuNP enabled high tumor accumulation by intravenously post-injection into SKOV-3 tumor bearing mice, which resulted in an intensive PA signal. Through integration with PTT, it was found that there was a very efficient anti-tumor activity, and no tumor recurrences. The results suggested that rGO-AuNP nanocomposite as an outstanding photothermal probe could be expected to widely applied in diagnostic and therapeutic studies of ovarian cancer.

## Data Availability Statement

All datasets presented in this study are included in the article/Supplementary Material.

## Ethics Statement

The animal study was reviewed and approved by The Ethics Committee of International Peace Maternity and Child Health Hospital, School of Medicine, Shanghai Jiaotong University, Shanghai, China.

## Author Contributions

ZW: investigation, validation, writing the manuscript, and funding acquisition. XS and TH: investigation and validation. JS: conceptualization, project administration, validation, and writing-review and editing. YW: funding acquisition, project administration, and writing-review and editing. All authors contributed to the article and approved the submitted version.

## Conflict of Interest

The authors declare that the research was conducted in the absence of any commercial or financial relationships that could be construed as a potential conflict of interest.
