# Supplementary material for: A Sandwich Nanostructure of Gold Nanoparticle Coated Reduced Graphene Oxide for Photoacoustic Imaging-Guided Photothermal Therapy in the Second NIR Window
Source: Front Bioeng Biotechnol. 2020 Jun 30;8:655. doi: 10.3389/fbioe.2020.00655 (PMC7338568; doi:10.3389/fbioe.2020.00655)
Supplement: Supplementary file 1 [file Data_Sheet_1.docx]

Supplementary Material


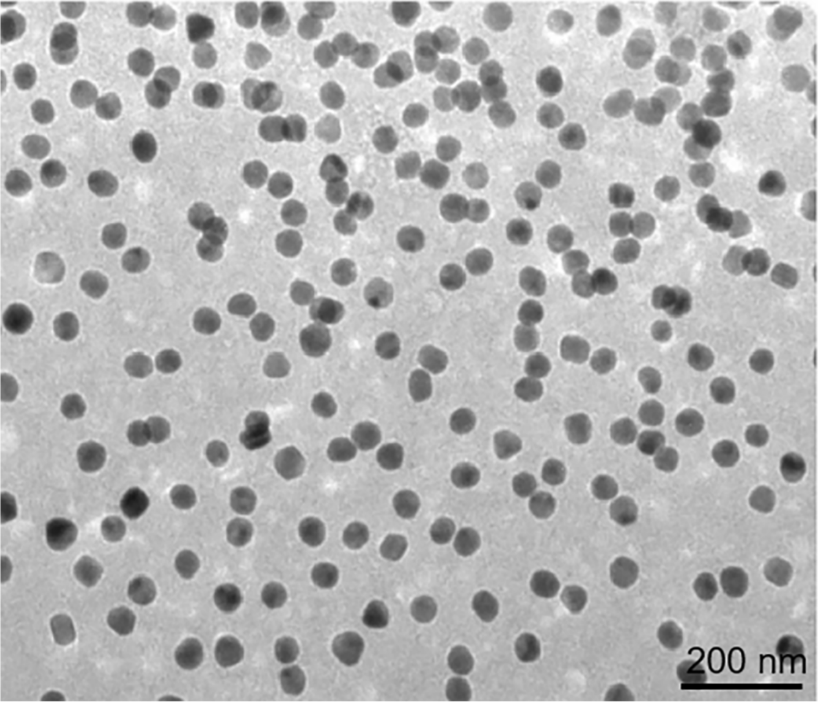


**Figure S1.** TEM images of the as-prepared AuNPs.


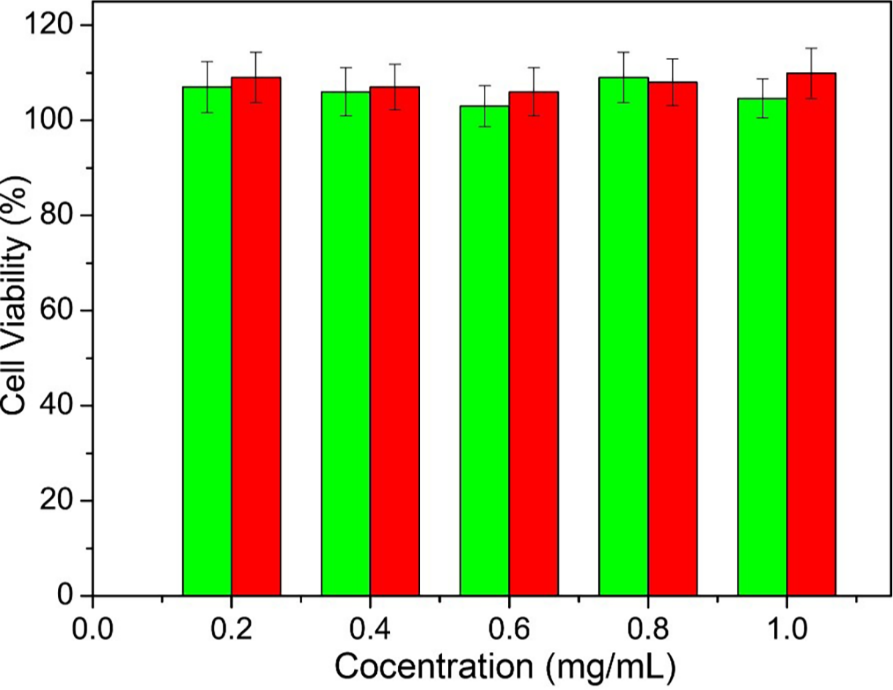


**Figure S2.** *In vitro* cell cytotoxicity of the rGO-AuNP (red column) and PBS (green column).


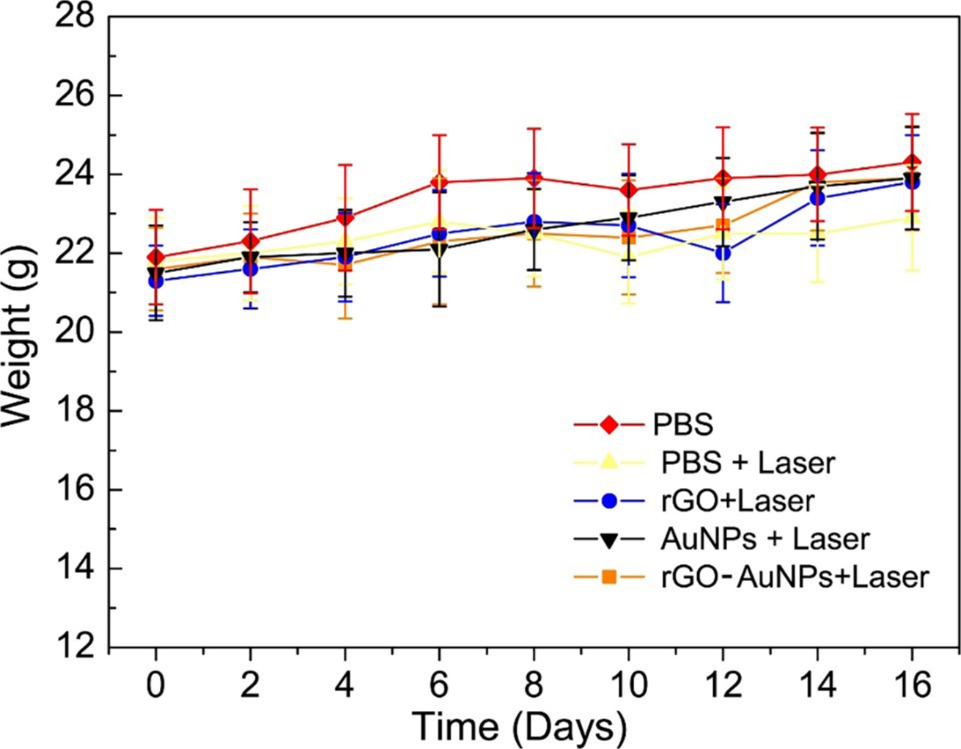


**Figure S3.** Body weights of the SKOV-3 tumor bearing mice after various treatments.
